# Metamaterial inspired electromagnetic bandgap filter for ultra-wide stopband screening devices of electromagnetic interference

**DOI:** 10.1038/s41598-023-40567-x

**Published:** 2023-08-16

**Authors:** Muath Al-Hasan, Mohammad Alibakhshikenari, Bal S. Virdee, Richa Sharma, Amjad Iqbal, Ayman A. Althuwayb, Francisco Falcone

**Affiliations:** 1 Department of Network and Communications Engineering, Al Ain University, 64141 Al Ain, United Arab Emirates; 2 Department of Signal Theory and Communications, Universidad Carlos III de Madrid, 28911 Leganes, Madrid Spain; 3 Center for Communications Technology, School of Computing and Digital Media, London Metropolitan University, London, N7 8DB UK; 4 Institut National de la Recherche Scientifique (INRS), Montreal, QC H5A1K6 Canada; 5 Department of Electrical Engineering, College of Engineering, Jouf University, 72388 Sakaka, Aljouf Saudi Arabia; 6 Department of Electric, Electronic and Communication Engineering and the Institute of Smart Cities, Public University of Navarre, 31006 Pamplona, Spain; 7 School of Engineering and Sciences, Tecnologico de Monterrey, 64849 Monterrey, Mexico

**Keywords:** Engineering, Electrical and electronic engineering

## Abstract

Presented here is a reactively loaded microstrip transmission line that exhibit an ultra-wide bandgap. The reactive loading is periodically distributed along the transmission line, which is electromagnetically coupled. The reactive load consists of a circular shaped patch which is converted to a metamaterial structure by embedded on it two concentric slit-rings. The patch is connected to the ground plane with a via-hole. The resulting structure exhibits electromagnetic bandgap (EBG) properties. The size and gap between the slit-rings dictate the magnitude of the reactive loading. The structure was first theoretically modelled to gain insight of the characterizing parameters. The equivalent circuit was verified using a full-wave 3D electromagnetic (EM) solver. The measured results show the proposed EBG structure has a highly sharp 3-dB skirt and a very wide bandgap, which is substantially larger than any EBG structure reported to date. The bandgap rejection of the single EBG unit-cell is better than − 30 dB, and the five element EBG unit-cell is better than − 90 dB. The innovation can be used in various applications such as biomedical applications that are requiring sharp roll-off rates and high stopband rejection thus enabling efficient use of the EM spectrum. This can reduce guard band and thereby increase the channel capacity of wireless systems.

## Introduction

Microwave structures can be assembled to exhibit electromagnetic bandgap (EBG) characteristics^1–3^. To be precise EBG structures are periodic and engineered to prevent or allow the propagation of electromagnetic (EM) waves in a specified band of frequency. Periodic structures when applied to RF/microwave planar transmission line waveguides can produce passband or stopband characteristics. By proper selection of the dimensions and periodicity of the structure the EM response of the structure can be controlled to either transmit or suppress certain signals to propagate through it^1,4^. This feature of EBG structures make them attractive in applications requiring EM shielding, e.g., medical equipment^5–14^.

EBG property can be implemented by periodically loading a microstrip transmission line with resonators or creating slots in the ground-plane of a dielectric substrate under a propagating microstrip line or stacking materials of different dielectric^15–17^. A transmission line can also display EBG property by periodically changing its impedance. In^9^, it’s shown that by tapering the profile of the low impedance sections in the transmission line will eliminate ripples in the passband, which is caused by the periodicity of the EBG structure. In^10–18^, it is shown that EBG can be established by locating shorted microstrip patches resembling mushroom like structures under the microstrip line. Another technique of creating EBG is through periodic reactive loading of a propagating microstrip line^19–24^. Periodic reactive loading reduces phase velocity (slow-wave effect), as compared to ordinary lines. This enhances the effective capacitance and/or inductance of the line^25^. In^26^, rectangular/sinusoidal variation of microstrip line width has been used to create periodicity^27^. However, in the case of variation in the width of the microstrip line require longer periods to exhibit EBG characteristics that result in a larger structure. In another technique periodic defects of different shapes are introduced in the ground plane that results in compact structures^22–24,26^. These defected ground structures offer challenges in the packaging. An alternate approach to induce reactive loading is to use shorted patch structures under the microstrip line^27^. The realization of these structures is carried out using a stack-up of three metal layers. In this structure the EBG property is created by the periodic variation of effective dielectric properties of the material. This methodology is compatible with GaAs/GaN monolithic microwave integrated circuit (MMIC) process, which makes it appealing for microwave and millimeter-wave applications.

In this paper a technique is described to realize ultra-wide bandgap using an EBG structure that does not have an impact on its physical size. This is achieved by employing the 2-D metamaterial concept. This is achieved by stacking five layers in the following order: metal–substrate–metal–substrate–metal. The top layer is microstrip transmission line, the second layer is dielectric substrate, the third layer is circular patches, the fourth layer is dielectric substrate, and the fifth layer is metal ground-plane. This implementation is consistent with GaAs/GaN MMIC processes which makes it viable for diverse applications such as biomedical applications operating over microwave and millimeter-wave spectrums. The circular patches are connected to the ground-plane through the second dielectric layer using via-holes. This configuration is commonly referred to as a mushroom-type structure. Ultra-wide bandgap is accomplished by incorporating metamaterial characteristics to this structure. This was realized by inserting a dielectric ring on the patch.

## Metamaterial inspired mushroom type unit-cell

Normally mushroom type EBG structures are made from square or rectangular patches that reactively load a common transmission line^28^. In EBG structures based on square and rectangular patch the dominant loading on the transmission line is essentially capacitive. The reactive loading can be easily modified by varying the dimensions of the patch. However, in both cases the bandgap realized is narrow and its transmission response can be plagued with significant sized ripples.

**Figure 1:**
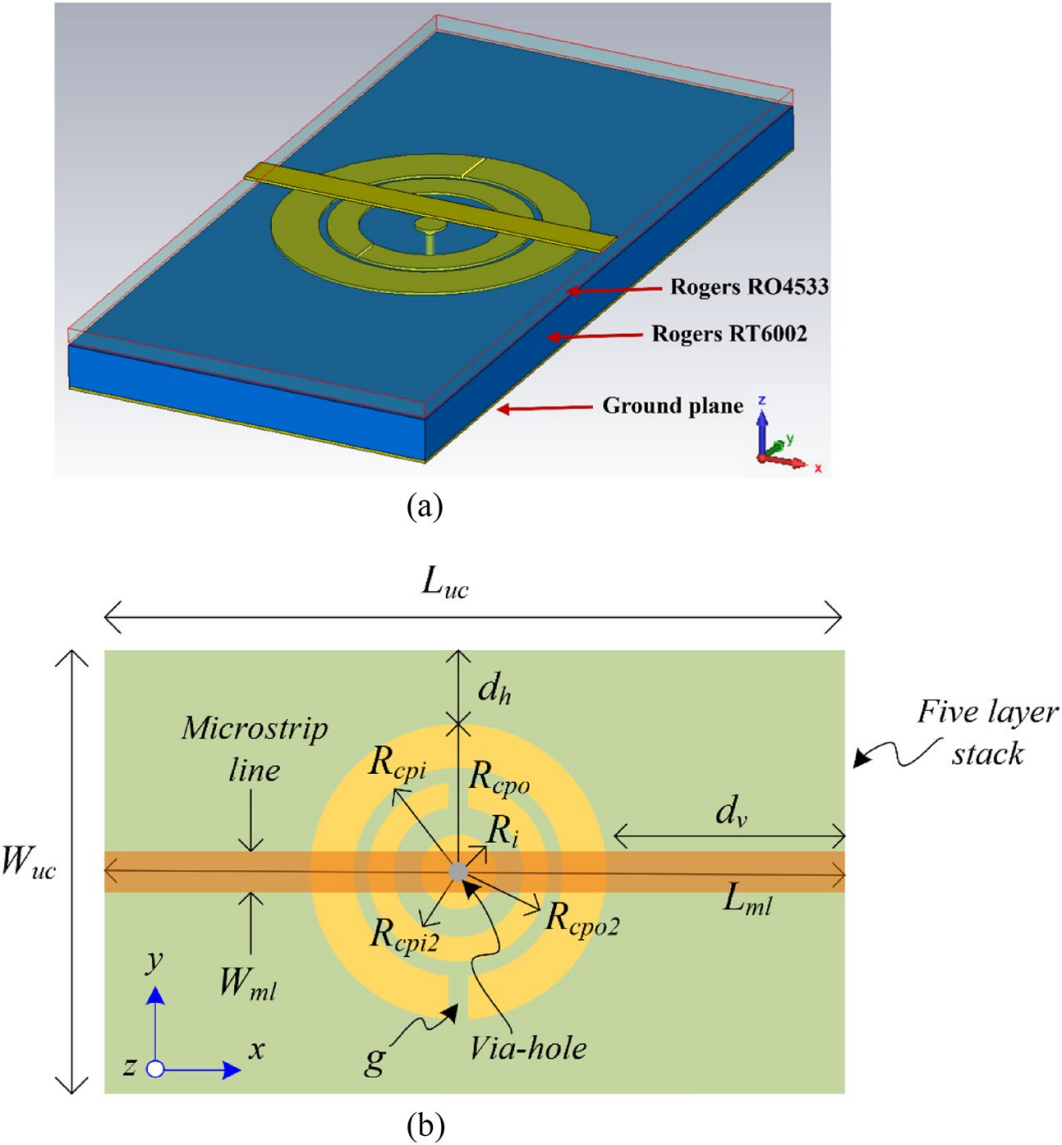
The proposed metamaterial inspired mushroom type EBG unit-cell, (**a**) isometric view, and (**b**) parameters defining the EBG unit-cell.

Figure 1 shows the implementation of the proposed metamaterial inspired mushroom type EBG unit-cell. The bandgap structure consists of a five-layer stack comprising bottom up from (1) metallic ground-plane, (2) dielectric substrate (Rogers RT6002), (3) microstrip circular patch, (4) dielectric substrate (Rogers RO4533), and (5) microstrip transmission line. The circular patch is connected to the ground-plane using via-hole through the Rogers RT6002 substrate. To realize ultra-wide bandgap performance two slit-ring resonators are embedded on the patch, as shown in Fig. 1a, which transform the unit-cell into a metamaterial structure. The parameters defining the EBG unit-cell are shown in Fig. 1b. The equivalent circuit model of the metallic via-hole is a shunt parallel *LC* circuit where inductance represents left-handed inductance, and the capacitance is represented by right-handed capacitance. The patch is capacitively coupled to the slit rings. This is modeled with a series *LC* circuit where the inductance is right-handed, and the capacitance is left-handed.

The circular patch of radius of ${R}_{i}$ is placed at the center of the unit-cell. The vertical and horizontal gaps between the circular patch and the unit-cell's edge are defined by ${d}_{h}$ and ${d}_{v}$, respectively. The unit-cell’s length $\left({L}_{uc}\right)$ and width $\left({W}_{uc}\right)$ can be expressed thus, ${{L}_{uc}=2(R}_{cp}+{d}_{v})$ and ${{W}_{uc}=2(R}_{cp}+{d}_{h})$. The metallic patch is connected to ground-plane through a via of radius and height of ${R}_{via}$ and ${h}_{via}$, respectively, which is located at the center of the circular patch. The length and width of the microstrip line are ${L}_{ml}$ and width ${W}_{ml}$, respectively, where ${L}_{ml}={L}_{uc}$. The EBG unit-cell structure was fabricated on commercially available substrates. The Rogers RT6002 substrate has a dielectric constant (${\varepsilon }_{r1}$) of 2.94, a thickness ${(h}_{1})$ of 0.6 mm, and loss-tangent of 0.0012. The Rogers RO4533 substrate has a dielectric constant (${\varepsilon }_{r2}$) of 3.45, a thickness ${(h}_{2})$ of 0.2 mm, and loss-tangent of 0.0025. The radius of the circular patch can be calculated using^29,30^

1 $$a=\frac{F}{{\left\{1+\frac{2h}{\pi {\varepsilon }_{r}F}\left[In\left(\frac{\pi F}{2h}\right)+1.7726\right]\right\}}^{0.5}}$$

2 $$F=\frac{8.791\times {10}^{9}}{{f}_{r}\sqrt{{\varepsilon }_{r}}}$$

where ${f}_{r}$ is the resonant frequency of the patch and $c$ is speed of light in free space. Note, $h$ and $a$ are in cm. Since fringing makes the patch electrically larger, the effective radius of patch is used and is given by^29,30^

3 $${a}_{eff}=a{\left\{1+\frac{2h}{\pi {a\varepsilon }_{r}}\left[In\left(\frac{\pi a}{2h}\right)+1.7726\right]\right\}}^{0.5}$$

The resonant frequency of the inner and outer split rings can be determined from Eqs. (4) and (5), respectively. These expressions were obtained from empirical studies.

4 $${f}_{i\_ring}=\frac{0.978c}{\left(2\pi {R}_{in}-g\right)\sqrt{{\varepsilon }_{r}}}$$

5 $${f}_{o\_ring}=\frac{1.028c}{\left(2\pi {R}_{out}-g\right)\sqrt{{\varepsilon }_{r}}}$$

where ${R}_{in}$ is the radius of the inner ring, and ${R}_{out}$ is the radius of the outer ring. ${R}_{in}=\left({R}_{cpi2}+{R}_{cpo2}\right)/2$ and ${R}_{out}=\left({R}_{cpi}+{R}_{cpo}\right)/2$. The structural parameters of the metamaterial inspired EBG unit-cell are listed in Table 1. For the parameter values given in Table 1, ${f}_{i\_ring}=23.2$ GHz and ${f}_{o\_ring}$=15.6 GHz. From empirical results the lower edge rejection frequency of the bandgap can be approximately defined by 0.74 ${f}_{o\_ring}$.

**Table 1:** Structural parameters (in millimeters) of the EBG unit-cell.

${L}_{uc}$	${W}_{uc}$	${h}_{1}$	${h}_{2}$	${h}_{via}$	${R}_{via}$	${L}_{ml}$	${W}_{ml}$	${R}_{i}$	${R}_{cpo}$	${R}_{cpi}$	${R}_{cpo2}$	${R}_{cpi2}$	${d}_{v}$	${d}_{h}$	$g$
6	6	0.6	0.2	0.6	0.05	6	0.5	0.2	2	1.4	1.3	0.9	1	1	0.05

**Figure 2:**
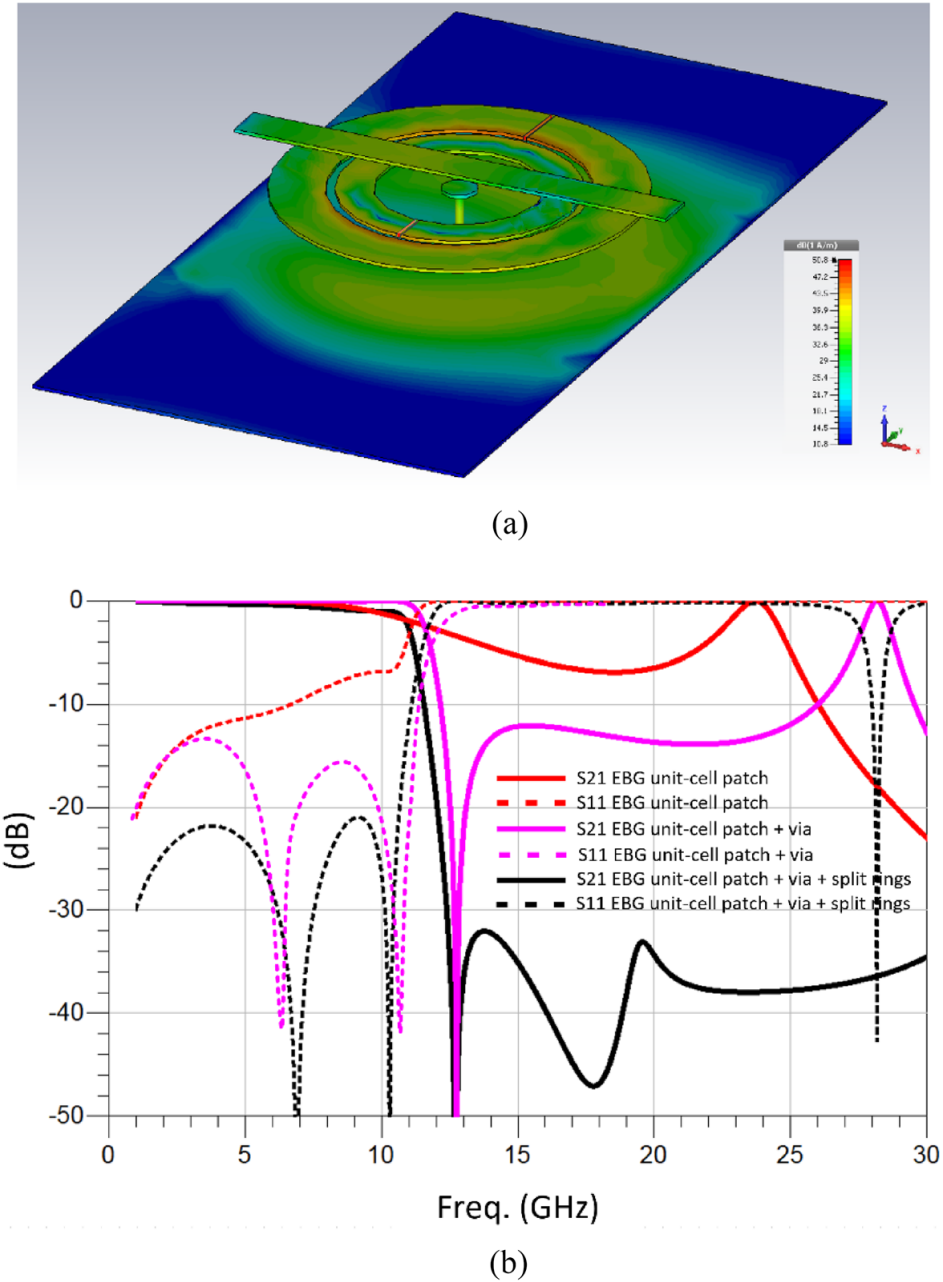
(**a**) Surface current density over the metamaterial inspired mushroom type EBG unit-cell at 11.5 GHz, and (**b**) *S*-parameter response of the metamaterial inspired mushroom type EBG unit-cell under three conditions, i.e., with no via-hole, with via-hole connection, and with via-hole and slit ring resonators.

The bandwidth of the EM bandgap is determined by the reactive loading of the ground-plane connected circular patch and associated split rings with the propagating microstrip line. When a microwave or millimeter-wave signal propagates over a microstrip transmission line its EM field will interact with the circular patch and the split rings which is located under it as illustrated in Fig. 1. The EM field will induce surface currents over the patch and split rings, as shown in Fig. 2a. The current density is greatest between the split rings. The currents at the center of the patch will find the shortest path to ground which is through the metallic via hole connecting the patch and the ground-plane. The reactive loading on the microstrip line is increased due to the split rings and the consequence of this is the bandgap is widened.

*S*-parameter analysis of the EBG unit-cell structure was carried out using 3-D full wave electromagnetic software by CST Microwave Studio. Figure 2 shows that when a metallic via-hole is connected to the circular patch the 3-dB roll-off of the stopband (S_21_) at 11.5 GHz becomes significantly sharper and the bandgap increases. Moreover, the bandgap rejection is better than − 10 dB up to 26 GHz. This is because the reactive loading offered to the patch is increased with the addition of the metallic via-hole. By including the split ring resonators, the bandgap rejection improves substantially. The bandgap extends above 30 GHz, and the bandgap rejection is better than − 30 dB. The return-loss (S_11_) is better than − 20 dB. This is because the inclusion of the split rings transforms the EBG unit-cell to a metamaterial and causes increased reactive loading to the transmission line.

## Equivalent circuit model of the proposed mushroom type EBG unit-cell

**Figure 3:**
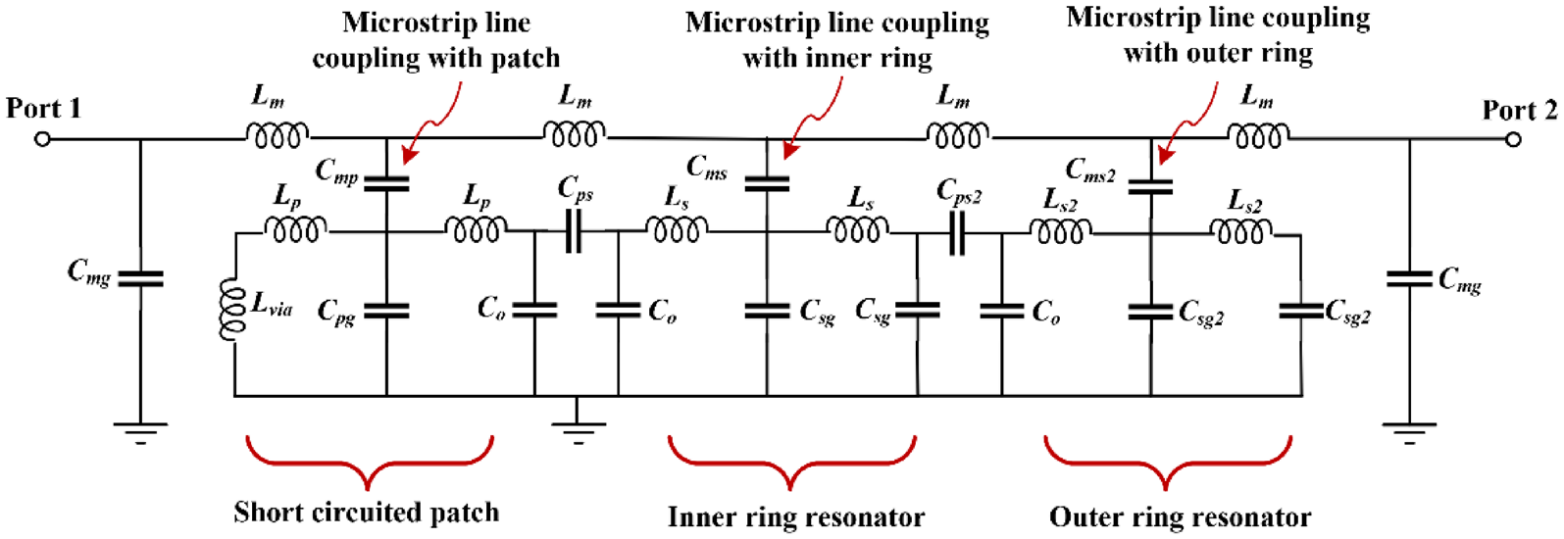
Equivalent circuit model of the proposed metamaterial inspired mushroom type EBG unit-cell.

**Figure 4:**
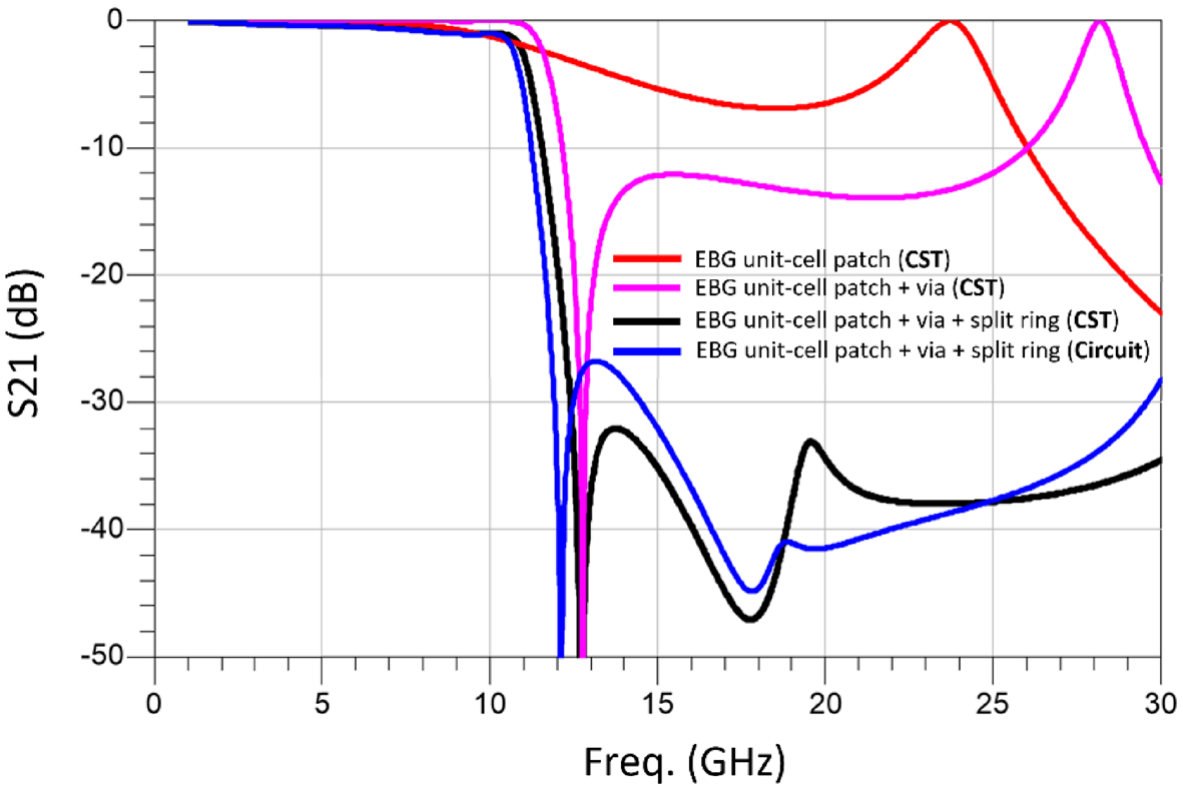
Insertion-loss response using CST Microwave Studio and the equivalent circuit model of the proposed EBG unit-cell.

Insight on the parameters that dictate the performance of the proposed metamaterial inspired mushroom type of EBG unit-cell can be obtained from its equivalent circuit model shown in Fig. 3. The circuit model approximates the physical structure of the unit-cell. The microstrip line is approximated by a lossless π-model having series inductance ($${L}_{m})$$ and shunt capacitance ($${C}_{mg}$$). Coupling between the microstrip line and the circular patch is through capacitance ($${C}_{mp})$$. The coupling between the microstrip line and the inner and outer split rings is through capacitances $${C}_{ms}$$ and $${C}_{ms2}$$, respectively. The magnitude of the coupling depends upon the microstrip’s length ($${L}_{ml}$$) and width ($${W}_{ml}$$) and the radii of the circular patch ($${R}_{cp})$$ and the split rings ($${R}_{i} \& {R}_{o})$$. The capacitance between patch and ground-plane is represented by capacitance $${(C}_{pg})$$. The circular patch has inductance ($${L}_{P})$$ and is capacitively coupled to the split rings through capacitances $${C}_{ps}$$ and $${C}_{ps2}$$. The open ends of the patch create parasitic fringe capacitance ($${C}_{o}$$) between patch and ground-plane. The capacitance between the inner and outer split rings and ground are $${C}_{sg}$$ and $${C}_{sg2}$$. The via-hole is modelled on a straight wire having inductance ($${L}_{via}$$) and radius ($${R}_{via})$$ and height ($${h}_{via}$$). The equivalent circuit model of the metallic via-hole is a shunt parallel *LC* circuit where inductances $${L}_{via}$$ and $${L}_{p}$$ represents left-handed inductance, and the capacitance $${(C}_{pg})$$ is represented by right-handed capacitance. The patch is capacitively coupled to the split rings of the patch through $${C}_{ps}$$ and $${C}_{ps2}$$. This coupling is modeled with a series *LC* circuit where the inductances $${L}_{s}$$ and $${L}_{s2}$$ are right-handed, and the capacitances $${C}_{sg}$$ and $${C}_{sg2}$$ are left-handed. The values of the equivalent circuit model components are given in Table 2. Figure 4 shows the insertion-loss (S_21_) response of the equivalent circuit model and that obtained from CST Microwave Studio. The Roll-off is 45 dB/GHz and bandgap range is 18 GHz from 11.5 to 30 GHz for rejection greater than 25 dB. There is excellent agreement between the equivalent circuit model and CST simulated response.

**Table 2:** Component values of the equivalent circuit model parameters.

${L}_{m}$ (nH)	${C}_{mg}$ (pF)	${C}_{mp}$ (pF)	${L}_{P}$ (nH)	${L}_{via}$ (nH)	${C}_{pg}$ (pF)	${C}_{ps}$ (pF)	${C}_{o}$ (pF)	${C}_{sg}$ (pF)	${C}_{sg2}$ (pF)	${L}_{s}$ (nH)	${L}_{s2}$ (nH)	${C}_{ms}$(pF)	${C}_{ms2}$(pF)
0.75	0.0475	0.27	0.205	0.1	0.19	0.15	0.047	0.6174	0.1933	0.1	0.05	4	0.954

## Finite periodic EBG bandstop filter based on the proposed EBG unit-cell

**Figure 5:**
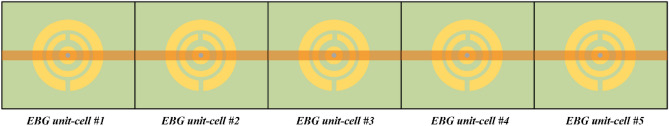
Top view of the proposed five element EBG structure.

**Figure 6:**
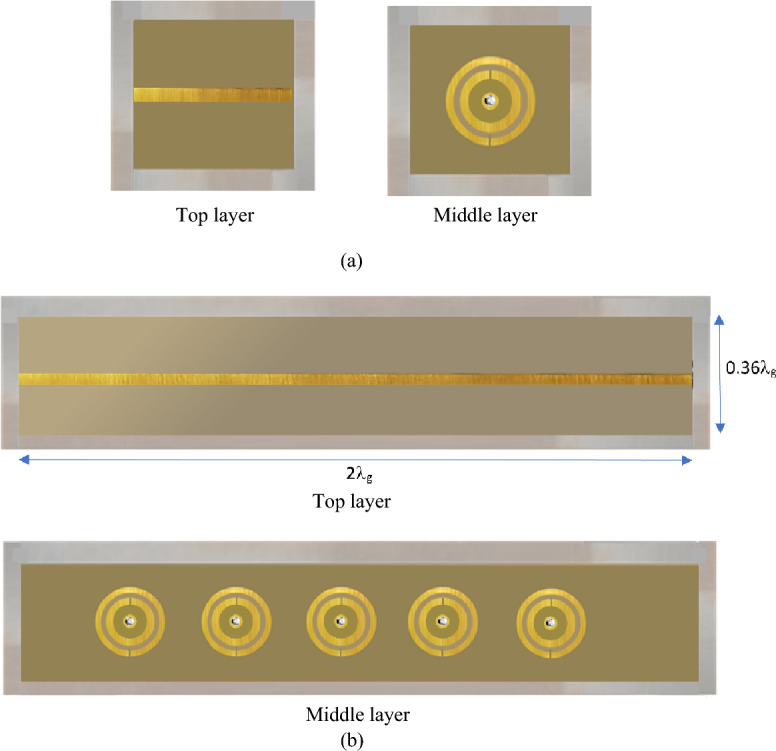
The fabricated 2-D bandstop filter based on the proposed metamaterial inspired mushroom type EBG unit-cell, (**a**) top transmission line and middle layer of a single unit-cell EBG structure, and (**b**) top transmission line and middle layer of the five unit-cell EBG structure where λ_g_ is at the 3-dB cut-off frequency of 12.5 GHz.

**Figure 7:**
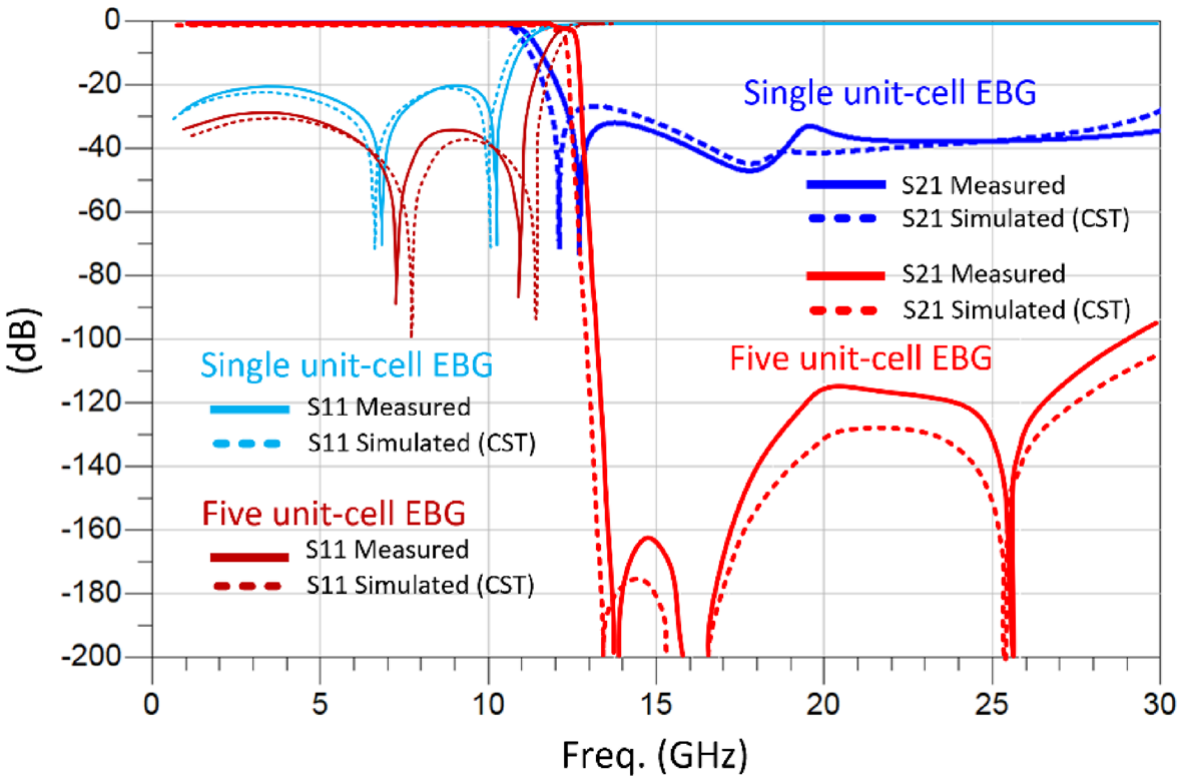
Simulated and measured S-parameters as function of frequency of the of the single and five EBG unit-cell.

A finite periodic EBG filter in Fig. 5 is based on the proposed metamaterial inspired EBG unit-cell. The filter is constructed by loading 50 $\Omega$ microstrip transmission line with five EBG unit-cells that are equally spaced. The filters were fabricated on dielectric substrate Rogers RT6002 and Rogers RO4533. The gap between the adjacent unit-cells is ${R}_{cp}/2$. The two substrates were glued together with a very thin layer of Eccobond 45 epoxy with a dielectric constant of 3.4. The overall dimensions of the EBG filter are 28 × 5 × 0.8 mm^3^. Figure 6 shows the fabricated 5-element EBG filter. Figure 7 shows the simulated and measured insertion-loss responses of the single and the five-element filter. The simulation was done using CST Microwave Studio. The insertion-loss was measured using a Network Analyzer. It is evident from the results the five unit-cell EBG provides a significantly sharper 3-dB roll-off and a bandgap with a rejection of approximately − 100 dB from 12 GHz to beyond 30 GHz. The response of the five-element filter is significantly superior to the single unit-cell EBG. This is due to the two layers of different dielectric constants used to construct the EBG unit-cells. When two different dielectric materials are combined in an EBG unit cell, it creates a more complex and structured arrangement. This combination can lead to an enhanced bandgap performance compared to a single-layer EBG structure. The presence of two dielectric layers introduces additional periodicity and resonances, resulting in wider and more effective bandgap regions. Moreover, each individual unit cell contributes to the overall suppression of specific frequencies, and the combination of multiple cells leads to a cumulative effect, resulting in a deeper bandgap. There is excellent agreement between the simulation and measured results.

In Table 3, the proposed EBG filter is compared with different EBG structures some of which have been recently reported. The comparison metrics includes size in guided wavelength, passband return-loss, the degree of attenuation in the bandgap, the 3-dB sharpness of the filter and the bandgap range. Compared to the cited works both the single unit-cell EBG structure is much smaller. The five unit-cell EBG structure has a significantly superior bandgap rejection any EBG reported to date. Although the proposed EBG structures have a highly sharp 3-dB roll-off, this is not as good as^35^. The shortcomings of^35^ are its poor return-loss response and limited bandgap range. The bandgap of the proposed EBG structures is substantially wider than any work reported to date.

**Table 3:** Performance comparison of the proposed metamaterial inspired EGB structure with published works.

References	EBG configuration	No. of unit cells	Length (${\lambda }_{g}$)	Passband return-loss (dB)	Bandgap rejection (dB)	Roll-off (dB/GHz)	Bandgap (GHz)/Freq. range for S_21_ > 25 dB
^1^	Tapered microstrip line	4	2.28	20	30	105	0.44/(0.75–1.19 GHz)
^31^	Tapered EBG with DGS*	3	0.67	13	32	10.9	6.0/(4–10 GHz)
^32^	Dumbbell-shaped DGS	3	0.87	5	40	56	2.2/(5.6–7.8 GHz)
^33^	Sinusoidal tapered line	6	1.69	3	47	87.5	6.0/(2.7–8.7 GHz)
^34^	Tapered dual plane EBG	6	2.30	8	35	14	5.1/(7.3–12.4 GHz
^35^	Shorted patch structure	8	1.34	3	40	235	1.9/(5.3–7.2 GHz)
This work	Metamaterial mushroom type EGB	1	0.46	20	30	192	> 18/(12–30 GHz)
This work	Metamaterial mushroom type EGB	5	1.77	30	95	192	> 17.7/(12.3–30 GHz)

*Defected ground structure.

## Conclusions

A novel electromagnetic bandgap structure is shown to exhibit desirable characteristics. Compared to state of the art the proposed five EBG unit-cell it is significantly smaller sizer, has the best passband return-loss, one of the highest bandgap rejections and the largest bandgap range reported to date. Even the bandgap range of the single EBG unit-cell is ultra-wide. These characteristics have been achieved by applying metamaterial properties to the mushroom type EBG unit-cell. This was achieved by embedding split ring resonators on the reactive loading comprising a circular patch which is short circuited to ground using a via hole. The patch and the split ring resonators are sandwiched between two dielectric substrates and is electromagnetically coupled to a microstrip transmission line implemented on the top side of the top substrate. The theoretical model of the EBG structure was verified using full-wave 3D electromagnetic solver from CST Microwave Studio.

## Data Availability

All data generated or analyzed during this study are included in this article.
